# Exendin-4 Promotes Survival of Mouse Pancreatic *β*-Cell Line in Lipotoxic Conditions, through the Extracellular Signal-Related Kinase 1/2 Pathway

**DOI:** 10.1155/2016/5294025

**Published:** 2016-08-30

**Authors:** Jianqiu Gu, Qian Wei, Hongzhi Zheng, Xin Meng, Jin Zhang, Difei Wang

**Affiliations:** ^1^Department of Endocrinology, The First Affiliated Hospital of China Medical University, 155 Nanjingbei Street, Heping District, Shenyang, Liaoning 110001, China; ^2^Department of Geriatric Endocrinology, The First Affiliated Hospital of China Medical University, 155 Nanjingbei Street, Heping District, Shenyang, Liaoning 110001, China

## Abstract

Type 2 diabetes is a heterogeneous disorder that develops as a result of relatively inappropriate insulin secretion and insulin resistance. Increased levels of free fatty acids (FFAs) are one of the important factors for the pathogenesis of type 2 diabetes and contribute to defective *β*-cell proliferation and increased *β*-cell apoptosis. Recently, glucagon-like peptide-1 (GLP-1) receptor agonists have been shown to possess an antiapoptotic effect, by increasing *β*-cell mass and improving *β*-cell function. However, their effects on *β*-cells in vitro against lipotoxicity have not been elucidated completely. In this study, we investigated whether the GLP-1 receptor agonist exendin-4 displays prosurvival effects in pancreatic *β*-cells exposed to chronic elevated FFAs. Results showed that exendin-4 inhibited apoptosis induced by palmitate in MIN6 cells. After 24 h of incubation, exendin-4 caused rapid activation of extracellular signal-related kinase 1/2 (ERK1/2) under lipotoxic conditions. The ERK1/2 inhibitor PD98059 blocked the antilipotoxic effect of exendin-4 on MIN6 cells. Exendin-4 also inhibited the mitochondrial pathway of apoptosis. This inhibition is associated with upregulation of BCL-2. Our findings suggested that exendin-4 may exert cytoprotective effects through activation of ERK1/2 and inhibition of the mitochondrial apoptosis pathway.

## 1. Introduction

Type 2 diabetes is a heterogeneous disorder characterized by peripheral insulin resistance, defects in insulin secretion, and *β*-cell apoptosis. Pancreatic *β*-cell dysfunction is central to the pathogenesis of type 2 diabetes, and the loss of functional *β*-cell mass in type 2 diabetes is at least in part secondary to increased *β*-cell apoptosis. Obesity is a well-known risk factor for diabetes and is characterized by elevated levels of circulating free fatty acids (FFAs) [1]. Prolonged exposure to elevated levels of FFAs has been shown to cause defective *β*-cell proliferation and increased *β*-cell apoptosis. Therefore, lipotoxicity plays an important role in underlying the mechanism of type 2 diabetes.

Glucagon-like peptide-1 (GLP-1), an incretin hormone secreted by intestinal L-cells, is a recent modality for the treatment of type 2 diabetes. GLP-1 and its receptor agonists exhibit a variety of benefits to diabetic patients. GLP-1 can be used against hyperglycemia and obesity in most T2DM diabetic patients by stimulating insulin secretion, inhibiting glucagon secretion, slowing gastric emptying, and promoting satiety [2]. Among all these effects, the major target of GLP-1 actions is the pancreatic *β*-cell [3–5]. GLP-1 has been found to promote *β*-cell proliferation [6, 7]. Furthermore, recent data have shown that GLP-1 and its receptor agonists promote the survival of *β*-cell lines when challenged with various apoptotic stimulators, including hyperglycemia, inflammatory cytokines, oxidative stress, and endoplasmic reticulum stress [8–10]. However, until now whether exendin-4, a GLP-1 receptor agonist, exerts cytoprotective effects in pancreatic *β*-cells under lipotoxic condition had not been determined completely.

Therefore, the aim of the present study was to investigate the potential antiapoptotic and prosurvival actions of exendin-4 in pancreatic *β*-cells under a chronic lipotoxic condition and the underlying signaling pathways involved.

## 2. Materials and Methods

### 2.1. Materials

Exendin-4 was purchased from Prospec (Rehovot, Israel). Fatty-acid-free bovine serum albumin (BSA, fraction V), palmitate, Hoechst33258, 3-(4,5-dimethylthiazol-2-yl)-2,5-diphenyltetrazolium bromides (MTT), and PD98059 were purchased from Sigma-Aldrich (St. Louis, MO, USA). The anti-phospho-ERK1/2 and anti-total ERK1/2 antibodies were obtained from Cell Signaling Technology (Danvers, MA, USA). The anti-BAX and anti-BCL-2 antibodies were obtained from Santa Cruz Biotechnology (Heidelberg, Germany). An enzyme-linked immunosorbent assay- (ELISA-) based bromodeoxyuridine (BrdU) incorporation kit was from Roche (Basel, Switzerland). The caspase-3 activity assay kit was from R&D Systems (Minneapolis, MN, USA).

### 2.2. Cell Culture

Mouse pancreatic *β*-cell line MIN6 was a kind gift from Biochemistry Lab of China Medical University with passage number of 10 to 20. They were maintained in Dulbecco modified Eagle medium (DMEM) containing 25 mM glucose, 15% fetal bovine serum (FBS), 100 U/mL penicillin, 100 mg/mL streptomycin, 100 mg/mL L-glutamine, and 5 *μ*g/L *β*-mercaptoethanol in a 37°C humidified atmosphere with 5% CO_2_ and 95% air. Cells at 80% confluence were washed with phosphate-buffered saline (PBS) and preincubated overnight in serum-free DMEM before treatment. Followed assays were conducted as designed.

### 2.3. Fatty Acid, Exendin-4, and ERK1/2 Inhibitor Treatment of MIN6 Cells

MIN6 cells were cultured in a high glucose (25 mM) serum-free DMEM with 0.5% BSA or 0.4 mM palmitate added 0.5% BSA, with or without ERK1/2 inhibitor, in the presence or absence of exendin-4. The 0.4 mM palmitate fatty acid solution was prepared as described previously [11]. Briefly, a 20 mM solution of palmitate in 0.01 M NaOH was incubated at 70°C for 30 min. Then 330 *μ*L 30% BSA and 400 *μ*L palmitate/NaOH mixture were mixed together, filter-sterilized, and added into 20 mL DMEM. The concentration of BSA was 0.5% in all medium. We selected a 25 mM glucose concentration for all tests because an increased glucose concentration was essential for MIN6 proliferation, and 25 mM glucose protected MIN6 cells from apoptosis [12]. Graded doses of exendin-4 (1, 10, 100, and 500 nM) were prepared freshly before tests. The ERK1/2 inhibitor used in the study was PD98059 (50 *μ*M).

### 2.4. Cell Viability Assay

Cell viability was assessed by MTT as described previously [13]. 5000 cells per well were set up onto 96-well plates. Before detection, cells were incubated with 1 mg/mL MTT for 1 h. The medium was removed and the formazan product was solubilized with 150 *μ*L dimethyl sulfoxide. Viability was assessed by spectrophotometry at 570 nm absorbance using a 96-well plate reader.

### 2.5. Cell Proliferation Assay

Proliferation of MIN6 cells was evaluated with an ELISA-based BrdU incorporation kit. Briefly, MIN6 cells were set up onto 96-well plates at 80% confluence and incubated overnight in serum-free DMEM before experiments, and BrdU was added to the culture medium for 1 h before cells harvest. Cells were then fixed and incubated with a peroxidase-conjugated anti-BrdU antibody, and immune complexes were quantified by spectrophotometry.

### 2.6. Hoechst33258 Assay

Apoptosis was evaluated by Hoechst33258 staining. The Hoechst positive cells represent the apoptotic cells, which were characterized with chromatin condensation or fragmented nuclear membrane. Briefly, Hoechst staining was performed by exposing the cell slides to 10 *μ*g/mL Hoechst33258 for 10 min at room temperature. The cells were counted by fluorescence microscopy after staining.

### 2.7. Caspase-3 Activity Assay

Caspase-3 activity was measured in triplicate with a Caspase-3 Colorimetric Assay kit, according to the manufacturer's manual. In brief, after two time washes with ice-cold PBS, cells were harvested and centrifuged at 10,000 rpm for 10 min, followed by the addition of 1 *μ*L dithiothreitol (DTT) and 100 *μ*L lysis buffer. Cell lysates were placed in a 96-well plate and incubated for 2 h at 37°C with 10 *μ*L Caspase-3 Colorimetric Substrate (DEVD-pNA). Absorbance at 405 nm was measured with a 96-well plate reader.

### 2.8. Western Blot

Western blotting was carried out as described previously [12]. Briefly, protein was extracted with a cell lysis buffer. 50 *μ*g protein samples (in 20 *μ*L buffer) were separated by SDS-electrophoresis on 12% gradient polyacrylamide gels and transferred onto PVDF membranes. The membranes were incubated with the primary antibodies with appropriate concentration, including anti-phospho-ERK1/2 antibody (1 : 1,000), anti-total ERK1/2 antibody (1 : 1,000), anti-BAX antibody (1 : 200), and anti-BCL-2 antibody (1 : 200). Immunodetection was performed with ECL advance, and resulting images were analyzed by Scion Image software version 4.0.3.2 (Scion Corporation, Frederick, MD).

### 2.9. Statistical Analysis

All data are presented as mean ± standard error (SE). Statistical analyses were performed with SPSS software version 13.0 (SPSS Inc., Chicago, IL) with statistical significant set at *p* < 0.05. Differences between groups were determined by one-way analysis of variance (ANOVA) followed by post hoc testing and correction by least significant difference/Dunnett's *t*-test. If the *F* ratio was statistically significant, a Turkey's post hoc test was considered.

## 3. Results

### 3.1. Exendin-4 Promotes MIN6 Cells Survival under Lipotoxic Condition

To determine the effects of exendin-4 on *β*-cell survival and proliferation, MIN6 cells were incubated in the 0.5% BSA (BSA) and 0.4 mM palmitate added 0.5% BSA (PA), with or without increasing concentrations of exendin-4 (1–500 nM) (Ex) for 24 h. Cell viability and proliferation were assessed by the MTT assay and an ELISA-based BrdU incorporation kit, respectively.

**Figure 1:**
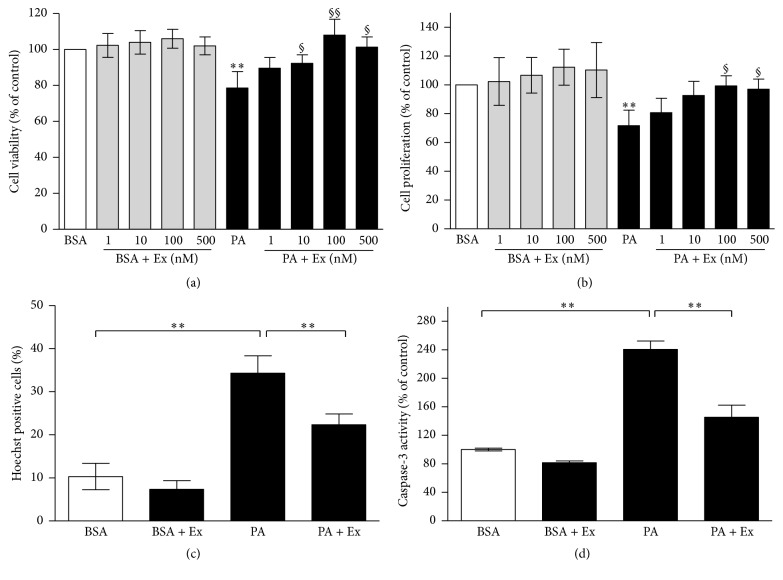
Exendin-4 inhibits apoptosis in palmitate-treated MIN6 cells. MIN6 cells were incubated in 0.5% BSA (BSA) or 0.4 mM palmitate added 0.5% BSA (PA), in the presence or absence of increasing concentrations of exendin-4 (1–500 nM) (Ex), in serum-free DMEM for 24 h. Exendin-4 was added at a concentration of 100 nM for Hoechst33258 staining and caspase-3 activity assays. (a) Cell survival was assessed by MTT assay. Cell survival results are expressed as percentage of control (cells treated with BSA alone) and represent the mean ± SE of five independent experiments. ^*∗∗*^ *p* < 0.01, versus BSA; ^§^ *p* < 0.05, ^§§^ *p* < 0.01, versus PA. (b) Cell proliferation was evaluated by BrdU incorporation kit. Cell proliferation results are expressed as percentage of control (cells treated with BSA alone) and represent the mean ± SE of five independent experiments. ^*∗∗*^ *p* < 0.01 versus BSA; ^§^ *p* < 0.05, ^§§^ *p* < 0.01, versus PA. (c) Apoptosis was evaluated by Hoechst33258 staining. Values are expressed as percentage of apoptotic cells and represent the mean ± SE of three independent experiments. ^*∗∗*^ *p* < 0.01. (d) Apoptosis was assessed by caspase-3 activity assay. Values are expressed as percentage of control (cells treated with BSA alone) and represent the mean ± SE of three independent experiments. ^*∗∗*^ *p* < 0.01.

Cell survival (Figure 1(a)) was significantly reduced by palmitate treatment (28%, *p* < 0.01 versus BSA). Exendin-4 treatment reversed palmitate-induced reduction of cell viability, and 100 nM exendin-4 displayed a major effect of restoring the cell viability by 27% (*p* < 0.01 versus PA). Exendin-4 treatment alone showed a nonsignificant promotion to cell survival, and 100 nM exendin-4 provided the best potentiation (112%, *p* = 0.21 versus BSA).

Cell proliferation (Figure 1(b)) was decreased under palmitate exposure (38%, *p* < 0.01 versus BSA). This decrease was inhibited by exendin-4 treatment, most obviously at 100 nM (29%, *p* < 0.01 versus PA). Exendin-4 treatment alone displayed a nonsignificant increase in cell proliferation, and 100 nM exendin-4 provided the greatest tendency (120%, *p* = 0.11 versus BSA). In the presence of PA, 100 nM exendin-4 achieved a significant proliferative effect (91%, *p* = 0.03 versus PA).

We next assessed cell apoptosis by Hoechst33258 assay and caspase-3 activity assay. For Hoechst33258 assay, MIN6 cells were incubated with or without 0.4 mM palmitate, in the presence or absence of 100 nM exendin-4 (Figure 1(c)). PA exposure for 24 h induced apoptosis (34.3%, *p* < 0.01 versus BSA), which was reversed by 100 nM exendin-4 treatment by decreasing the apoptosis to 11.9% (*p* < 0.01, PA + Ex versus PA). Similar results were found using caspase-3 activity assay (Figure 1(d)). Apoptosis was significantly increased in cells treated with PA alone (133%, *p* < 0.01, PA versus BSA). 100 nM exendin-4 treatment with PA presence resulted in a significant decrease of apoptosis (87%, *p* < 0.01, PA + Ex versus PA).

### 3.2. Exendin-4 Exerts Antilipotoxic Effects through Phosphorylation of ERK1/2

**Figure 2:**
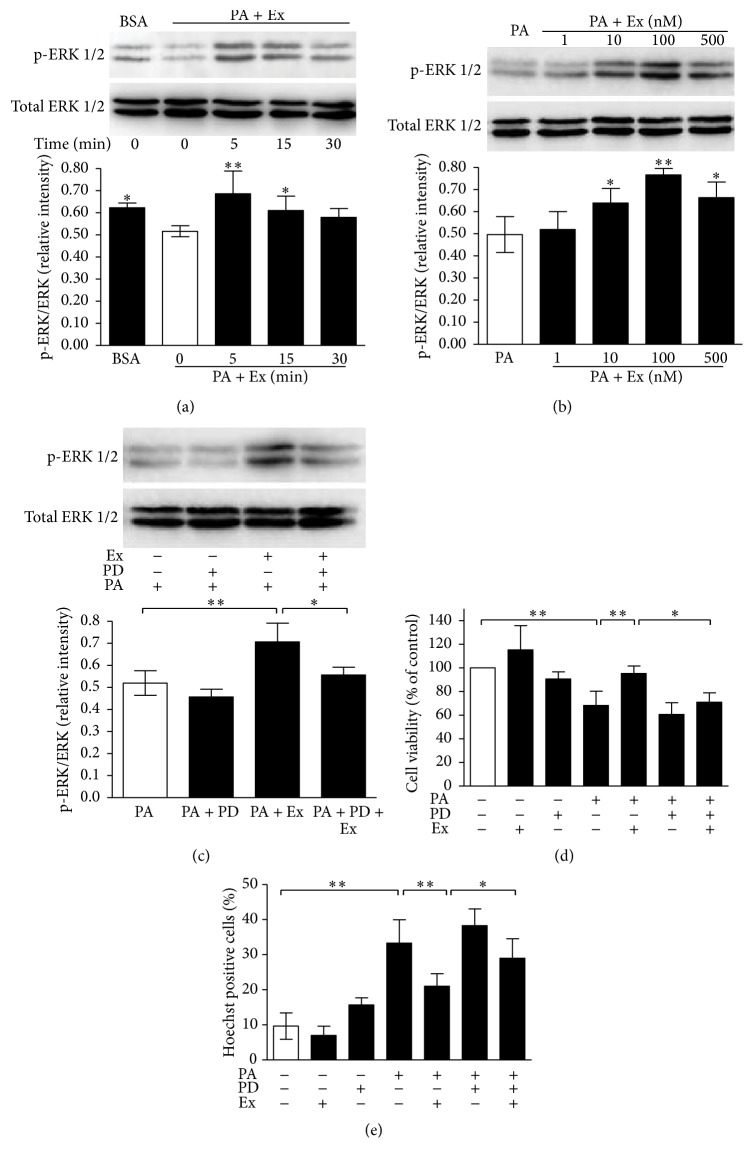
The antilipotoxic effects of exendin-4 on cell survival and apoptosis involve ERK1/2 pathway. MIN6 cells were preincubated overnight in serum-free DMEM and then incubated in serum-free DMEM containing 0.5% BSA (BSA) or 0.4 mM palmitate added 0.5% BSA (PA) for 24 h. Increasing concentrations (1–500 nM) of exendin-4 (Ex) were used to treat the cells for the indicated times. (a) Total ERK1/2 and p-ERK1/2 protein expression after 0–30 min of treatment with 100 nM Ex. Values are expressed as relative intensity ratio and represent the mean ± SE of three independent experiments. ^*∗*^ *p* < 0.05, ^*∗∗*^ *p* < 0.01, versus PA + Ex at 0 min. (b) Total ERK1/2 and p-ERK1/2 protein expression after 5 min of treatment with increasing concentrations (1–500 nM) of Ex. Values are expressed as relative intensity ratio and represent the mean ± SE of three independent experiments. ^*∗*^ *p* < 0.05, ^*∗∗*^ *p* < 0.01, versus PA group without Ex. (c) Total ERK1/2 and p-ERK1/2 protein expression after 5 min of treatment with 100 nM Ex, with or without inhibitor. Before Ex stimulation, cells with PA were pretreated for 30 min with ERK1/2 inhibitor, 50 *μ*M PD98059 (PD). Values are expressed as relative intensity ratio and represent the mean ± SE of three independent experiments. ^*∗*^ *p* < 0.05, ^*∗∗*^ *p* < 0.01. (d) Cell survival was assessed by MTT assay. ^*∗*^ *p* < 0.05, ^*∗∗*^ *p* < 0.01. (e) apoptosis assessed by Hoechst33258 staining. MIN6 cells were incubated with BSA or PA, with or without PD, in the presence or absence of 100 nM Ex, in serum-free medium for 24 h. Values of survival are expressed as percentage of control (cells treated with BSA alone) and represent the mean ± SE of five independent experiments. Values of apoptosis are expressed as percentage of apoptotic cells and represent the mean ± SE of 10 random fields of vision of three independent experiments. ^*∗*^ *p* < 0.05, ^*∗∗*^ *p* < 0.01.

We investigated the effect of exendin-4 on ERK1/2 phosphorylation under palmitate treatment, by detecting the ratio of phosphorylated ERK1/2 expression to total ERK1/2 expression (Figure 2(a)). The ERK1/2 phosphorylation was blocked by palmitate exposure (0.624 ± 0.048 versus 0.496 ± 0.062, *p* < 0.05 BSA versus PA + Ex at 0 min). At the end of the preincubation period, 100 nM exendin-4 was added. Cells were collected at indicated time points after that. Phosphorylation of ERK1/2 was increased by exendin-4 treatment in a time-dependent manner. The maximal effect was observed at 5 min (0.721 ± 0.135 versus 0.496 ± 0.062, *p* < 0.01 PA + Ex at 5 min versus PA + Ex at 0 min).

Exendin-4 also induced the phosphorylation of ERK1/2 in a concentration-dependent manner (Figure 2(b)) and 100 nM exendin-4 treatment produced the most effective potentiation (0.744 ± 0.083 versus 0.494 ± 0.117, *p* < 0.01 PA + Ex at 100 nM versus PA).

To establish the induction of phosphorylation of ERK1/2 by exendin-4, we did further treatment using PD98059, a specific ERK1/2 inhibitor (Figure 2(c)). The exendin-4-induced phosphorylation of ERK1/2 was obviously suppressed by PD98059 (0.707 ± 0.096 versus 0.556 ± 0.050, *p* < 0.05 Ex + PA versus Ex + PD + PA), whereas the effect of PD98059 on ERK1/2 phosphorylation without exendin-4 was similar to that of PA alone (0.459 ± 0.057 versus 0.519 ± 0.071, *p* = 0.217 PA + PD versus PA).

We also determined the role of the ERK1/2 inhibitor on the cytoprotective effect of exendin-4 by MTT assay and Hoechst33258 assay (Figures 2(d) and 2(e)). Consistent with the aforementioned results, exendin-4 treatment promoted cell survival (95.3 ± 3.7% versus 68.4 ± 6.9%, *p* < 0.01 Ex + PA versus PA) and prevented apoptosis of MIN6 cells (21.2 ± 2.1% versus 33.5 ± 3.7%, *p* < 0.01 Ex + PA versus PA) under lipotoxic condition, whereas PD98059 suppressed this promotion of cell survival (71.0 ± 4.6% versus 95.3 ± 3.7%, *p* < 0.05 Ex + PD + PA versus Ex + PA) and attenuated the restore of apoptosis (29.2 ± 3.2% versus 21.2 ± 2.1%, *p* < 0.05 Ex + PD + PA versus Ex + PA) under lipotoxic condition.

All these results strongly suggested that exendin-4 protected MIN6 cells against lipotoxicity, at least in part, via activation of ERK1/2 signaling pathway.

### 3.3. Antiapoptotic Effect of Exendin-4 Involves the Mitochondrial Apoptosis Pathway

**Figure 3:**
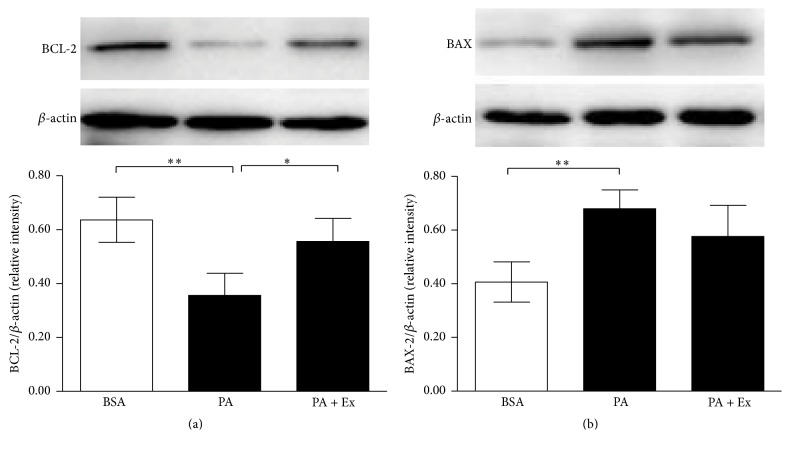
The antiapoptotic effect of exendin-4 involves the mitochondrial apoptosis pathway. MIN6 cells were incubated in serum-free DMEM with 0.5% BSA (BSA) or 0.4 mM palmitate added 0.5% BSA (PA), with or without 100 nM exendin-4 (Ex) for 24 h. Protein expression of BCL-2 (a) and BAX (b) was assessed by western blot in MIN6 cells. Values are expressed as relative intensity ratio of BCL-2 or BAX to *β*-actin and represent the mean ± SE of three independent experiments. ^*∗*^ *p* < 0.05, ^*∗∗*^ *p* < 0.01.

Western blot analysis of BCL-2 and BAX were conducted after 24 h culture under lipotoxic condition (Figure 3). We found a significant decreased expression of the antiapoptotic protein BCL-2 (Figure 3(a), *p* < 0.01 versus BSA) and enhanced expression of the proapoptotic protein BAX (Figure 3(b), *p* < 0.01 versus BSA) in MIN6 cells under palmitate treatment. While the exendin-4 treatment showed a significant increased expression of BCL-2 (Figure 3(a), *p* < 0.05 versus PA) and a nonsignificant decreased expression of BAX levels (Figure 3(b), *p* = 0.22 versus PA), compared with the parallel lipotoxic condition cultured without exendin-4.

## 4. Discussion

The incretin glucagon-like peptide-1 (GLP-1), the major gastrointestinal product of proglucagon processing, has been shown to exert trophic effects on *β*-cells [14]. Exendin-4 is a GLP-1 receptor agonist with various capabilities including cellular protection [15]. Although roles of exendin-4 on cell proliferation and antiapoptosis have been demonstrated in *β*-cells, its mechanisms are not fully understood. Moreover, whether exendin-4 protects *β*-cells against lipotoxicity remains to be determined. In the present study, we found that exendin-4 inhibited palmitate-induced apoptosis via activating ERK1/2 pathway and also through inhibiting mitochondrial apoptosis pathway in the MIN6 cells.

Lipotoxicity and glucotoxicity play vital parts in type 2 diabetes [16–18]. Physiologic levels of lipids and glucose are not toxic but essential to *β*-cell function, while prolonged exposure of pancreatic *β*-cells to high levels of fatty acid impairs insulin gene expression [19], inhibits insulin synthesis and secretion [20], and induces *β*-cell apoptosis [11, 21, 22]. In vivo, the normal plasma concentration of FFA is less than 0.6 mmol/L but could exceed 1 mmol/L in type 2 diabetic patients [23, 24]. Palmitate and oleate are the two most represented FFAs in plasma (20 to 25% each), linked to albumin in the circulation [25, 26]. To mimic what is observed in vivo, we used a mixture of palmitate with 5% fatty-acid-free BSA. This high albumin concentration was used to obtain an appropriate albumin to nonesterified fatty acid ratio. In our study, the direct effects of long-term elevated palmitate on the survival of pancreatic *β*-cells were studied. We showed that palmitate markedly enhanced apoptosis and attenuated cell survival of MIN6 cells. Consistent with previous observations [27, 28], theoretically, antilipotoxicity through blockade of apoptosis and enhancement of proliferation may be a novel approach to protect pancreatic *β*-cells.

Accumulating evidence supports the role of GLP-1 mimetics and enhancers in regulating proliferation and protecting from cellular apoptosis. These agents constitute a novel class of antidiabetes medications, which might have a major impact on the treatment of type 2 diabetes [29–31]. In this study, we demonstrated that treatment with exendin-4, a GLP-1 agonist, protected pancreatic *β*-cell from palmitate-induced toxicity by enhancing proliferation and inhibiting apoptosis, in a dose-dependent manner (Figure 1). In addition, exendin-4 treatment activated the ERK1/2 signaling pathway.

ERK1/2, a well-known downstream target of Raf-1, has been shown to participate in pathways controlling *β*-cell survival. It has been reported in several studies that acute glucose and protein kinase A signaling may regulate *β*-cell growth and survival through ERK1/2 [32–34]. On the other hand, others have shown that activation of ERK1/2 is required for human islet apoptosis in response to chronic exposure to high glucose concentrations or interleukin-1*β* [35]. Thus, ERK1/2 signaling is activated in both prosurvival and proapoptotic conditions. The outcome may depend on the timing and duration of ERK1/2 activation [36]. In the present study, our findings showed that exposure of MIN6 cells to chronic lipotoxic conditions reduced the phosphorylation of ERK1/2. In addition, this reduction was reversed by exendin-4 treatment through a rapid activation of ERK1/2. Further treatment with the ERK1/2 inhibitor PD98059 blocked this protective effect of exendin-4 (Figure 2). These data indicate that exendin-4 exerted cytoprotective effect on pancreatic *β*-cells against lipotoxicity, at least in part via the ERK1/2 pathway.

The mechanisms underlying apoptosis are regulated by defined biochemical pathways, involving members of the BCL-2 family [37]. In mammalian cells, the BCL-2 family of proteins plays a central role in modulating mitochondrial-dependent apoptosis. Both prosurvival and proapoptotic BCL-2 family members are critical death regulators via mitochondrial apoptosis pathway [38]. Among BCL-2 family proteins, prosurvival BCL-2 stabilizes the mitochondrial membrane and prevents the cytochrome C release from the mitochondria and the followed activation of caspases. In the present study, BCL-2 was found to be downregulated and the proapoptotic BAX was upregulated, which was in accordance with the increased activity of caspase-3 under lipotoxic condition. After exendin-4 treatment, we observed that these alterations were partially counteracted (Figure 3). So, our results indicate that exendin-4 conducts its protective effect through inhibiting mitochondrial apoptosis pathway, by promoting BCL-2 protein expression. This finding may provide further insight into the mechanisms of cytoprotective effect of exendin-4.

Interestingly, some BCL-2 family proteins were found to be involved in Erk1/2 signaling pathway. As reviewed by Cagnol and Chambard [39], proapoptotic proteins BAD and BAX were found to be activated by Erk1/2 activation. In contrast, antiapoptotic proteins BCL-2 and BCL-Xl were downregulated by ERK1/2 activation. On the other hand, phosphorylation of proapoptotic protein BAD was assumed to be involved in ERK1/2 promoted cell survival [40]. In human pancreatic cancer cells, inhibition of ERK1/2 activities caused downregulation of antiapoptotic proteins BCL-2, MCL-1, and BCL-Xl without affecting the proapoptotic proteins BAX and BAK [41]. These various reports might be interpreted depending on different cell types or conditions. In our study, we found both ERK1/2 activation and enhanced BCL-2 expression induced by exendin-4 treatment under lipotoxic condition. Further study on the relation between ERK1/2 activation and inhibition of mitochondrial apoptosis pathway may reveal more detailed molecular mechanism of GLP-1 mediated cytoprotective role on pancreatic *β*-cells. Another defect of our study is the MIN6 cell line we used. MIN6 is an insulinoma cell line derived from a C57BL/6 transgenic mouse [42]. Compared to other insulin-secreting cell lines, MIN6 cells express GLUT-2 and glucokinase and display a similar glucose stimulating insulin secretion to normal primary *β*-cells [43]. The insulin secretion of MIN6 cells reaches the maximal level approximately sevenfold above the basal level at 25 mmol/L [42], which is consistent with the performance of our MIN6 cells (data not shown). But as an immortalized cell line, some of its characters, especially the proliferative specific, are not exact same with primary *β*-cells. This could be a confounding factor within any study on this aspect. So further confirmatory study using primary *β*-cells is needed.

## 5. Conclusion

In conclusion, it is likely that exendin-4 induced ERK1/2 activation is an important pathway, by which exendin-4 exerts antiapoptotic actions against palmitate-induced lipotoxicity. Besides, the potential cytoprotective effect of exendin-4 on *β*-cells involves the mitochondrial apoptosis pathway, by upregulating BCL-2. Because lipotoxicity is supposed to be responsible for *β*-cell loss in the pathological process of obesity-associated type 2 diabetes, the antilipotoxic effects on residual *β*-cells of exendin-4 may arouse interest, considering its therapeutic utilization in type 2 diabetes treatment.
